# Tyrosine Kinase Inhibitors as a Treatment of Symptomatic CNS Metastases in Oncogene-Driven NSCLC

**DOI:** 10.1155/2020/1980891

**Published:** 2020-09-03

**Authors:** Omer Gal, Elizabeth Dudnik, Ofer Rotem, Inbar Finkel, Idit Peretz, Alona Zer, Jacob Mandel, Alexandra Amiel, Tali Siegal, Jair Bar, Anastasiya Lobachov, Shlomit Yust

**Affiliations:** ^1^Neuro-Oncology Unit, Davidoff Cancer Center, Rabin Medical Center, Beilinson Campus, Petah Tikva 49100, Israel; ^2^Sackler Faculty of Medicine, Tel Aviv University, Tel-Aviv 69978, Israel; ^3^Thoracic Cancer Service, Davidoff Cancer Center, Rabin Medical Center, Beilinson Campus, Petah Tikva 49100, Israel; ^4^Baylor College of Medicine, 7200 Cambridge Suite 9a, Houston, Texas 77030, USA; ^5^Thoracic Oncology, Institute of Oncology, Sheba Medical Center, Tel HaShomer, Ramat Gan 5262000, Israel

## Abstract

Central nervous system (CNS) metastases occur frequently in oncogene-driven non-small cell lung cancer (NSCLC). Standard treatment approaches can potentially delay systemic treatment (surgical intervention) or result in neurocognitive impairment (radiotherapy). Recently, next-generation tyrosine kinase inhibitors (TKIs) have demonstrated remarkable intracranial activity. However, most clinical trials did not enroll patients suffering neurological symptoms. Our study aimed to assess the CNS activity of targeted therapies in this patient population. We present a case series of nine NSCLC patients with either *EGFR* mutation or *ALK* rearrangement and symptomatic CNS metastases that were treated with TKIs. Clinicopathological characteristics, treatment, and outcomes were analyzed. Most patients presented with symptomatic CNS metastases at time of metastatic disease presentation (6/9). Additionally, the majority of patients had leptomeningeal disease (6/9) and multiple parenchymal metastases. Patients presented with a variety of CNS symptoms with the most common being nausea, vomiting, headache, and confusion. Most patients (6/9) responded rapidly both clinically and radiographically to the targeted treatment, with a marked correlation between systemic and intracranial radiographic response. In conclusion, upfront use of next-generation TKIs in patients with oncogene-driven NSCLC with symptomatic CNS metastases is associated with reasonable intracranial activity and represents a valuable treatment option.

## 1. Introduction

Central nervous system (CNS) metastases occur in 24–44% of patients with advanced non-small cell lung cancer (NSCLC) [1]. Incidence of CNS metastases is even higher (24–58%) among patients with tumors harboring an epidermal growth factor receptor (*EGFR*) mutation, anaplastic lymphoma kinase (*ALK*) rearrangement, or c-*ROS* oncogene 1 (ROS1) rearrangement [2–8]. Brain metastases negatively affect survival and quality of life [9].

Until recently, the standard approach to the treatment of brain metastases was primarily local and included options such as surgery, whole brain radiation therapy (WBRT), and stereotactic radiosurgery (SRS). A frequent shortcoming of the local strategies is the necessity to delay systemic treatment which can be crucial in patients with rapidly progressing tumors [10]. Additionally, radiation therapy often can result in long-term complications, such as cognitive decline or radiation necrosis [11, 12]. Finally, WBRT has recently been demonstrated to have no impact on overall survival or quality of life [13].

Systemic approach to CNS metastases has been recently implemented into the treatment scheme of NSCLC patients. Initially, systemic approach included the use of platinum-based chemotherapy in patients with small asymptomatic brain metastases [10, 14, 15]. Later on, it has been demonstrated that platinum-based chemotherapy can be used as an upfront single modality treatment without compromising overall survival not only in asymptomatic patients, but also in patients with significant neurological symptoms [16]. Most recently, significant advances have been made in the field of immune check-point blockade and targeted therapy resulting in the implementation of these classes of agents in the treatment of patients with CNS metastases [17–22].

Specifically, in tumors harboring genomic alterations in *EGFR*, *ALK*, and *ROS1* genes, systemic treatment with tyrosine kinase inhibitors targeting each of the above listed abnormalities is associated with remarkable intracranial activity [19, 23]. For instance, treatment with osimertinib, a 3rd-generation *EGFR* tyrosine kinase inhibitor (TKI), in patients with *EGFR*-mutant tumors, is associated with intracranial objective response rate (ORR) of 54%–90% in various clinical settings [7, 24, 25]. Alectinib, a 2nd-generation *ALK* TKI, has demonstrated an intracranial ORR of 79% in treatment-naïve [26] and 64% in crizotinib-refractory *ALK*-rearranged NSCLC patients [19]. Treatment with another 2nd-generation *ALK* TKI, brigatinib, is associated with an intracranial ORR of 83% in treatment-naïve [27] and 67% in crizotinib-refractory patients [28]. Importantly, similar intracranial ORR with alectinib and brigatinib were seen in patients with active brain metastases and patients who received brain irradiation before treatment initiation, confirming the excellent CNS penetration of these novel compounds [19, 26, 28]. Treatment with a 3rd-generation *ALK* inhibitor, lorlatinib, has demonstrated an intracranial ORR of 87%, 55%, and 53% in patients previously treated with crizotinib, one non-crizotinib *ALK* TKI, and 2-3 non-crizotinib *ALK* TKIs, respectively, all either with or without previous chemotherapy [29]. According to the results of a combined analysis of 3 prospective clinical trials assessing entrectinib, a potent *ROS1* TKI, in *ROS1*-rearranged tumors, intracranial ORR with entrectinib was 55% in *ROS1* TKI naïve patients [30].

However, the vast majority of clinical trials assessing intracranial activity of targeted therapies only allowed enrollment of patients free from neurological symptoms [7, 19, 24–31]. The data regarding the intracranial activity of molecularly targeted agents in patients with neurological symptoms is limited. Our study aimed at assessing the CNS activity of targeted therapies in patients with NSCLC harboring targetable genomic alterations and brain metastases producing significant neurological symptoms.

## 2. Materials and Methods

The lung cancer clinic databases of two tertiary centers (Davidoff Cancer Center and Sheba Medical Center) were searched for patients with either *EGFR* mutation or *ALK* rearrangement and symptomatic BM that were treated with systemic therapy. Nine patients who fulfilled these criteria were included in our study. Demographic and clinical data including age, sex, treatment history, time to development of brain metastases, number of brain metastases, response to treatment (both radiographic and clinical), neurological symptoms, and survival were collected. The study was approved by the Rabin Medical Center IRB committee (0391-14-RMC).

## 3. Results

Table 1 summarizes the clinical data of the 9 patients (8 female, 1 male) with either *EGFR* mutation or *ALK* rearrangement (with targetable alterations) NSCLC who were treated with 1st-line TKI for symptomatic brain metastases. Median age at diagnosis was 72 years (range 51–85). All patients presented with stage IV disease with dissemination to metastatic sites, except one patient (patient 7) who presented with localized disease and had a brain-only relapse two years after primary definitive therapy. Most patients had symptomatic BM at metastatic disease presentation, with only 2 patients without CNS involvement and one with asymptomatic BM sequentially developing BM. Symptomatic presentation of BM was diverse and most patients suffered multiple symptoms, with the most common being nausea and vomiting, headache, and confusion. A majority of the patients (6 patients) had leptomeningeal disease, often accompanied by multiple (>10) parenchymal metastases. Leptomeningeal disease was diagnosed by brain imaging or cerebrospinal fluid cytology or both.

Table 2 summarizes treatment and response data, as well as subsequent treatment lines. Most patients (5/9) were treated with osimertinib, either as primary systemic therapy or as second-line therapy after failing another TKI. One patient with *ALK* rearrangement was treated with lorlatinib after failing on 3 previous treatment lines. The remaining 3 patients received either gefitinib or afatinib as first-line treatment. The majority of the patients (6 patients) responded rapidly to the targeted treatment, with marked correlation between systemic and intracranial response allowing for discontinuation or reduction in dexamethasone dose. Two patients were treated with antiseizure medications (Keppra 500 mg BID and 250 mg BID). Only one patient had seizures, did not respond to osimertinib, and rapidly succumbed to metastatic disease. Patient 7 did not have relevant systemic outcome since she had brain-only spread.

Three patients (patients 2, 3, and 8) had rapid disease progression on the targeted treatment and subsequently did not manage to perform radiographic follow-up. Two patients had brain-only progression. Both were treated with WBRT, and one of them was also treated with double-dose osimertinib. Patient 4 had massive systemic progression and was switched from afatinib to osimertinib. Patient 9 had progression in both the lung and the brain and did not receive subsequent active treatment.

Detailed descriptions of two patients are given in Table 2.

### 3.1. Patient 1

**Figure 1:**
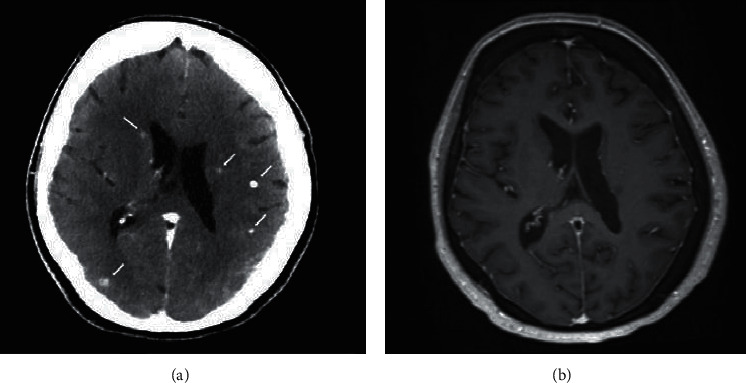
Dynamics of brain metastases on lorlatinib. Axial brain CT showing brain metastases (white arrows) after lorlatinib dose reduction (a). Axial brain MRI (T1 with contrast) performed two months after resuming full dose lorlatinib showing complete resolution of all brain lesions (b).

Patient 1 is a 59-year-old lady with metastatic *ALK*-rearranged lung cancer. Before BM diagnosis she was treated with crizotinib, ceritinib, and chemotherapy (carboplatin/pemetrexed) with partial systemic response. Approximately 1 year before presenting with symptomatic BM, while being treated with chemotherapy, she had asymptomatic intracranial progression with multiple BM and was switched to lorlatinib 100 mg/day, with rapid intracranial complete response (CR). However, due to peripheral neuropathy attributed to drug toxicity, the dose was decreased to 75 mg/day and later on to 50 mg/day which she took intermittently. Shortly after the last dose reduction, she presented to the ER with new sensory aphasia and headache and brain CT scan revealed new extensive intracranial dissemination (Figure 1(a)). Lorlatinib was promptly resumed at full dose (100 mg/day), with full symptomatic resolution and CR in the following MRI (Figure 1(b)). The response lasted 1.5 years. Unfortunately, her headaches recurred and brain MRI demonstrated new BM including leptomeningeal disease. She was treated with WBRT and continued treatment with lorlatinib due to good systemic control.

### 3.2. Patient 7

**Figure 2:**
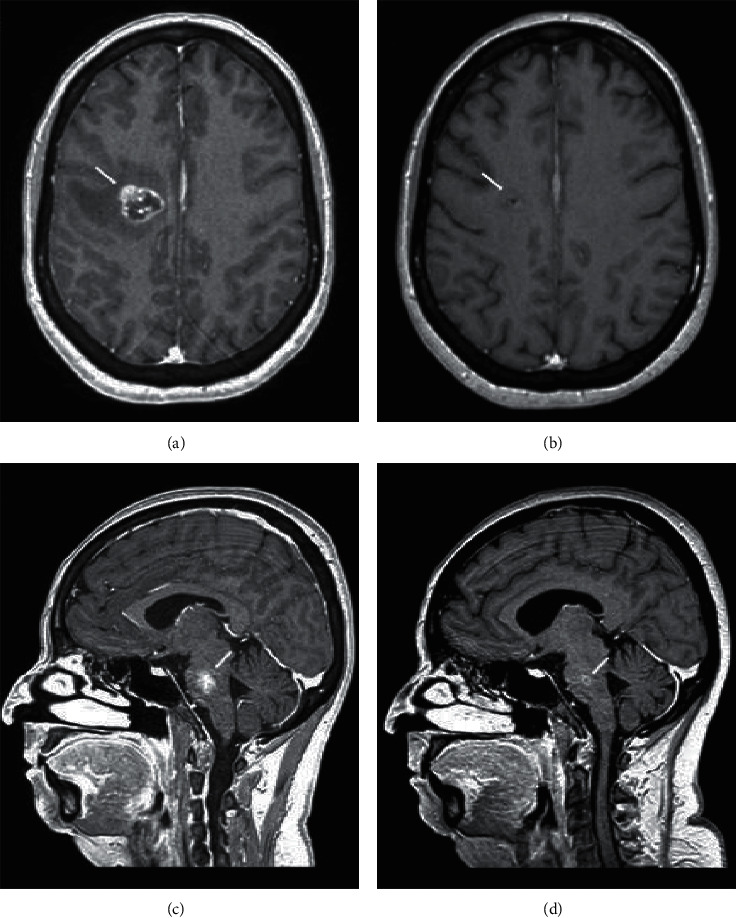
Dynamics of brain metastases on osimertinib. Brain MRI (T1 with contrast, axial, and sagittal) showing brain metastases (white arrows) before treatment (a and c) and 1 month after initiating treatment (b and d).

Patient 7 is a 52-year-old lady, who presented with simultaneous early stage left lung cancer (IIb) and bilateral hormone receptor and HER2 positive breast cancer (Ia). She underwent bilateral lumpectomy and lobectomy and received adjuvant treatment including chemotherapy (carboplatin + paclitaxel), anti-HER2 treatment (trastuzumab), and radiotherapy. After completing treatment, there was no evidence of disease, and she was started on adjuvant hormonal therapy (letrozole) and routine follow-up. Two years after diagnosis, she experienced weakness and nausea and later on also developed dizziness and gait disturbance. Brain MRI revealed 2 BM (right frontal and brainstem, Figures (2(a) and 2(c)), while systemic workup revealed no evidence of disease outside the brain. Molecular workup of her original lung tumor showed an *EGFR* exon 19 deletion. She was started on osimertinib 80 mg/day with rapid clinical improvement (within 2 weeks). Subsequent MRI scans showed significant response (Figures 2(b) and 2(d)) in both foci. Due to the fast and complete resolution of symptoms, she continued osimertinib with no focal treatment. Until the last follow-up, 9.8 months after diagnosis of symptomatic BM, the patient continues to be free of neurological symptoms and there is no evidence of systemic disease.

## 4. Discussion

Our case series illustrates the value of targeted therapy in the treatment of patients with oncogene-driven NSCLC and symptomatic CNS metastases. It gives an estimate on intracranial activity of these drugs in symptomatic intracranial disease. It also correlates the intracranial activity with the systemic activity of these compounds, focuses on failure patterns, and describes the whole course of the disease.

According to our data, *EGFR* TKIs and *ALK* TKIs achieved an excellent intracranial objective response in six out of nine treated patients, whereas the radiographic response was also accompanied by symptomatic relief. Importantly, the majority of the responses were durable and lasted 4.2–19 months since treatment initiation. Almost all of the cases of intracranial failure were rescued by brain radiotherapy or another systemic treatment. Finally, there was a concordance between systemic and intracranial responses.

This observation is in line with the previously reported data. For instance, in the series reported by Lin et al. and comprised of eighteen evaluable patients with *ALK*-rearranged NSCLC and symptomatic (eight patients) or large (≥1 cm; 10 patients) CNS metastases, intracranial ORR with alectinib was 72% and median intracranial duration of response was 17.1 months (95% CI, 14.3: not evaluable); all eight patients with symptoms attributable to CNS metastases had clinical improvement upon starting alectinib therapy [32]. Hochmair et al. reported on five patients with NSCLC harboring an activation mutation in the *EGFR* gene and symptomatic brain metastases achieving a complete and long-lasting intracranial remission with afatinib [33]. Additionally, Park et al. reported on overall ORR of 83% and median brain radiotherapy-free interval of 12.6 months (95% CI, 7.6–17.6) with gefitinib and erlotinib in patients with *EGFR*-mutant NSCLC and brain metastases, whereas 15 patients enrolled in the study (54% of the study population) had some neurological symptoms at baseline [34]. Finally, BRAIN trial comparing icotinib, an *EGFR* TKI, with upfront WBRT and platinum-based chemotherapy included 26 symptomatic patients (13 of them in the icotinib arm) and suggested similar benefits from icotinib—regardless of the presence or absence of neurological symptoms at baseline (HR for intracranial progression-free survival (PFS) of 0.57 (95% CI, 0.21–1.53) and 0.59 (95% CI, 0.35–0.99) for symptomatic and asymptomatic patients, respectively) [35]. BRAIN trial demonstrated median intracranial PFS of 10.0 months (95% CI, 5.6–14.4) with icotinib. Moreover, similar systemic (52%) and intracranial (65%) ORR with icotinib were observed [35].

All the abovementioned observations validate the upfront use of *EGFR* TKIs and *ALK* TKIs in patients presenting with symptomatic CNS metastases. This strategy represents a valuable treatment option allowing early initiation of systemic treatment, deferral of brain radiotherapy, and prevention of the long-term radiation-associated toxicity. Moreover, this approach represents an alternative to WBRT which is the only possible localized treatment option for patients with multifocal and large-volume intracranial disease that is not amenable for SRS/surgery, such as big parenchymal metastases or leptomeningeal spread. It should be mentioned that our study, similarly to the studies of Byeon et al. and Jiang et al. [36, 37], mainly includes multifocal large-volume intracranial disease in which no apparent benefit exists with the addition of radiotherapy to targeted treatment. However, it remains questionable whether the combined approach of targeted therapy with SRS is superior to targeted therapy alone in cases with oligometastatic disease in the brain.

It is important to emphasize the difference between the first-generation and the next-generation TKIs in terms of the ability to cross the blood-brain barrier and, as a result, the difference in their intracranial activity. For instance, cerebrospinal fluid (CSF)-to-plasma ratio of only 0.0006–0.0026 has been reported for crizotinib, the first *ALK*/*ROS1* TKI that has got regulatory approval for clinical use in *ALK*-rearranged and *ROS1*-rearranged tumors [38, 39]. This pharmacokinetic phenomenon represents the main reason for the impaired control of the disease in the CNS whenever crizotinib is used in the treatment of these tumor subtypes. Next-generation TKIs were specifically designed to effectively penetrate the blood-brain barrier and, therefore, have higher CSF-to-plasma ratio (for instance, one paper reported on CSF-to-plasma ratio for alectinib of 0.86) [40], whereas another one reported on lower values of 0.001–0.003 [41]. From the clinical perspective, higher blood-brain barrier penetration results in higher intracranial disease control rates and lower rates of intracranial disease progression with next-generation TKIs. For instance, in the ALEX study, intracranial ORR in *ALK*-rearranged patients who did not receive brain radiotherapy was 78.6% with alectinib and 40.0% with crizotinib, and CNS duration of response was NR (95% CI, 13.4–NR) with alectinib and 3.7 months (95% CI, 2.3–5.5) for crizotinib [26]. Similarly, in the FLAURA trial, intracranial ORR and median intracranial PFS with osimertinib (a 3rd-generation *EGFR* TKI) and 1st-generation *EGFR* TKIs in *EGFR*-mutant tumors were 91% and 68% (odds ratio for intracranial ORR-4.6; 95% CI, 0.9–34.9; *p*=0.066) and NR (95% CI, 16.5 months: not calculable) and 13.9 months (95% CI, 8.3 months: not calculable) (HR for intracranial PFS-0.48; 95% CI, 0.26–0.86; *p*=0.014), respectively [24]. Although our series could not address the differences in the intracranial activity between the first-generation and the next-generation TKIs in patients with neurological symptoms, based on the abovementioned data in asymptomatic patients, it seems preferable to use the novel compounds in symptomatic patients as well.

## 5. Conclusions

In conclusion, upfront use of next-generation *EGFR* TKIs and *ALK* TKIs in patients with *EGFR*-mutant and *ALK*-rearranged NSCLC with brain metastases is of value in multifocal, large-volume, and symptomatic intracranial tumors; their use in patients with symptomatic CNS metastases is associated with reasonable intracranial activity and symptomatic improvement. It remains to be seen whether the combined approach of targeted therapy with SRS is superior to targeted therapy alone in cases with small-volume oligometastatic disease in the brain.

## Figures and Tables

**Table 1:** Clinical and disease characteristics of patients included in the study.

Patient	Age	Gender	Additional metastatic sites (not including brain)	Molecular aberration	Time to symptomatic BM (months)	BM symptoms	BM number/characteristics
1	59	Female	LN, liver, bone, adrenal	*ALK* rearrangement	33	Aphasia, headache	>10, LMD
2	62	Female	Lung, LN	*EGFR* L747S mutation	22	Gait disturbance, N/V	>10, LMD
3	78	Female	Lung, LN	*EGFR* L858R mutation	73	Headache, seizure	7
4	75	Female	Lung, liver, bone, adrenal	*EGFR* exon 19 deletion	0	Confusion	4, LMD
5	82	Female	Bone	*EGFR* L858R mutation	0	Confusion, cognitive decline	LMD
6	72	Male	LN, liver, bone	*EGFR* exon 19 deletion	0	N/V	>10, LMD
7	51	Female	None	*EGFR* exon 19 deletion	0	Gait disturbance, N/V, dizziness	3
8	85	Female	Adrenal	*EGFR* exon 19 deletion	0	Dizziness	LMD
9	55	Female	Lung, adrenal	*EGFR* L861Q mutation	0	Headache, N/V, confusion, vision loss	>10

BM: brain metastases; LN: lymph nodes; *ALK*: anaplastic lymphoma kinase; *EGFR*: epidermal growth factor receptor; N/V: nausea and vomiting; LMD: leptomeningeal disease.

**Table 2:** Treatment and outcome data.

Patient	Treatment before symptomatic BM	Treatment with symptomatic BM	Best systemic response	Best radiographic intracranial response	Neurological symptomatic response	Steroids (D) dose before, after treatment	TTP (months)	Progression site	Subsequent lines	Survival from symptomatic BM diagnosis or last follow-up
1	Crizotinib, ceritinib, carboplatin/pemetrexed	Lorlatinib	CR	CR	Complete resolution	10 mg, 0 mg	19	Stable	None	19 (alive)
2	Afatinib	Osimertinib	NA	NA	Deterioration	16 mg, NA	2.1	Brain	WBRT	2.8
3	Gefitinib	Osimertinib	NA	NA	Deterioration	12 mg, NA	2.3	NA	None	2.3
4	None	Afatinib	PR	PR	Clinical improvement	6 mg, NA	4.2	Lung, LN, liver	Osimertinib	5.9
5	None	Gefitinib	PR	PR	Clinical improvement	NA, NA	15.8	NA	None	15.8
6	None	Osimertinib	PR	PR	Complete resolution	8 mg, 0 mg	10.2	Brain	WBRT, HD osimertinib	15.1
7	None	Osimertinib	NA	PR	Complete resolution	4 mg, 0 mg	Stable (9.6 FU)	Stable	None	9.6 (alive)
8	None	Afatinib	PR	NA	Deterioration	10 mg, 10 mg	3.3	Brain, lung	None	3.3
9	None	Osimertinib	PR	PR	Clinical improvement	6 mg, 4 mg	Stable (9.5 FU)	Stable	None	9.5 (alive)

BM: brain metastases; D: dexamethasone; TTP: time to progression; CR: complete response; NA: not applicable; PR: partial response; FU: follow-up, month; LN: lymph nodes; WBRT: whole brain radiation therapy; HD: high dose.

## Data Availability

The data used to support the findings of this study are available on request from the corresponding author. The data are not publicly available due to privacy or ethical restrictions.
